# Universality out of order

**DOI:** 10.1038/s41467-022-29955-5

**Published:** 2022-04-29

**Authors:** Petter Holme

**Affiliations:** 1 Department of Computer Science, Aalto University, 02150 Espoo, Finland; 2 Center for Computational Social Science, Kobe University, Kobe, 657-8501 Japan

**Keywords:** Complex networks, Interdisciplinary studies

Orders, rankings, and hierarchies on one side, universal statistical laws on the other—it is rare that these core concepts of complex systems science meet. This Comment sets the scene for some recent discoveries in this too seldomly visited border zone.

## An example: pecking orders

In the first decades of the last century, Norwegian schoolboy Thorleif Schjelderup-Ebbe spent his summer vacation trips to a farm watching chickens^1^. He noticed that when hens peck on each other in a fight, they follow specific patterns. If hen A pecked on hen B, B would not peck on A. It didn’t necessarily mean that A was stronger, bigger, or older than B. Instead, it seemed like they settled on who was above when they first met and then kept that order unless some rare incident reversed it^2^.

**Fig. 1:**
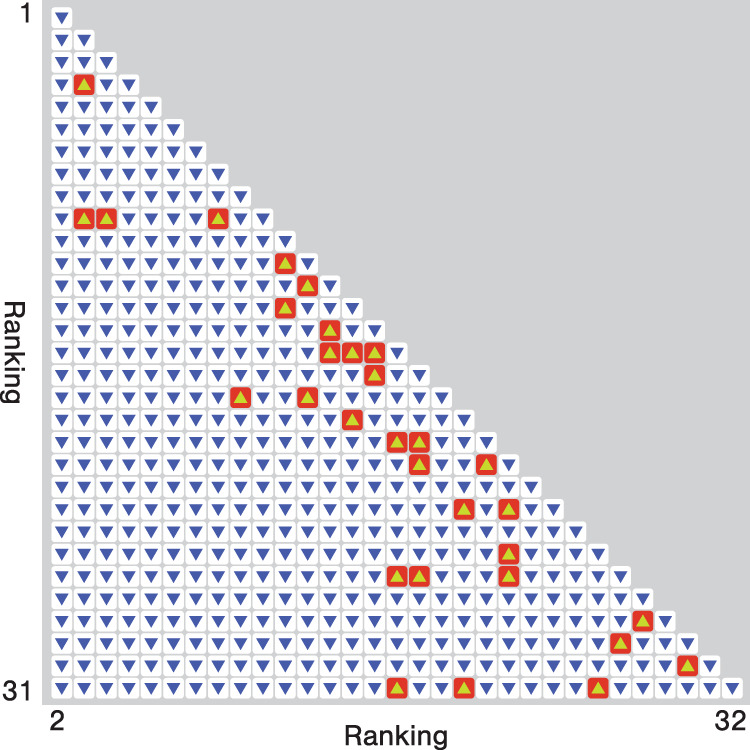
An illustration of dominance relations within a group of Leghorn hens (*Gallus gallus domesticus*) observed in 1946^3^. (Data available at http://konect.cc/networks/moreno_hens/) The rows and columns correspond to the identification numbers of individual hens. Arrows show the dominance relations—an arrow at row *i* and column *j* points down if *i* dominates *j*, and up if *j* dominates *i*. The ranking is obtained by a simple greedy relabeling (i.e., not necessarily the one minimizing the number of discordant pairs with respect to the ranking—a so-called “minimum violation ranking”). As typical for real-world rankings of many kinds, we can see that pecking orders of hens are not complete, linear rankings^1^.

In 1913, at the tender age of 19, Schjelderup-Ebbe published his first scientific paper about these observations^2^. Four decades later, the concept of pecking order had entered common knowledge. Even though Schjelderup-Ebbe reported that there could be cycles within the dominance structures, pecking orders have become archetypes of social rankings. This illustrates just how prone humans are to spot rankings—the prototypical example can, strictly speaking, not be ordered completely from top to bottom. Figure 1 shows the pecking order observed in ref. ^3^.

## Rankings as simplified representations of hierarchies

Rankings are everywhere in the human world, for business and pleasure. It seems like a profoundly human exercise to rank things—for teleological reasons^4^ or otherwise—as we do it even when the precise order lacks significance. Iñiguez et al. mention that rankings can serve as a way to simplify complex situations^5^. Probably the most typical structures that people simplify to rankings are hierarchies. Hierarchies and rankings are closely related and occasionally conflated. Pecking orders, for example, belong to the more general research theme of “dominance hierarchies.”

Hierarchy is one of those concepts whose imprecision seems to beget its popularity. There are many, more or less problem-specific, definitions of hierarchy. These are, furthermore, often inconsistent^6^. To most authors, hierarchies are collections of levels, from top to bottom. Members of the hierarchy would inherit the ranking of their levels—so that one could order members of different levels, but not (necessarily) members of the same level.

Herbert Simon—probably the most-quoted authority on hierarchical organization—points at the following relationship between hierarchy and ranking: “The world is a large matrix of interactions in which most of the entries are very close to zero, and in which, by ordering those entries according to their orders of magnitude, a distinct hierarchic structure can be discerned”^7^. Hierarchies were the basis for Simon’s theory of complex systems^8^, which continues to influence our understanding of large-scale systems to this day.

## The many meanings of universality

With the Enlightenment and the advent of social statistics came the idea that society could obey laws akin to Newtonian mechanics. Kant, for example, asserted that no matter if free will exists or not, “human actions, like every other natural event are determined by universal laws. However obscure their causes, […] we may be able to discern a regular movement in it, and that what seems complex and chaotic in the single individual may be seen from the standpoint of the human race as a whole [as] steady and progressive”^9^. Such statistical laws of the human world often take the form of power-law relations, where the exponent is universal, independent of specific details about the systems. Perhaps the most well-known examples are Zipf’s law—stating that the frequency of the *k*th most common word is inversely proportional to *k* (i.e., having an exponent of –1)—and the Pareto principle of wealth distribution^10^.

In the physical theory of critical phenomena, the meaning of universality came to change in the 1960s and 70 s. The critical exponents, describing the phase transitions, seemed to fall into a few so-called universality classes^11^. Describing how systems change with the length scale—the renormalization group theory—led to a theoretical foundation for this scarcity of critical exponents. Moreover, it motivated statistical physicists to search for universal laws outside of physical systems^12^. It became a prominent example of how universality can appear from emergence—another defining feature of complex systems.

## Universality in ranking dynamics

As we have seen so far, ranking dynamics, universality, and criticality are close to the heart of physics-flavored complexity science. It seems obvious that the border area of these ideas would be a fertile ground for discovery. Given that, Iñiguez et al.^4^. extends a surprisingly small collection of studies.

Blumm et al.^13^. took a bona fide physics approach—defining a microscopic model to describe how the fluctuation of the rank of items depends on the rank. They found that the items could belong to one of three phases depending on their fitness and the system’s noise level. This situation is notably different from the traditional dynamic critical phenomena in physics, where every unit belongs to the same phase^14^. Rather, it resembles the localization of epidemic phases in heterogeneous networks^15^.

Like Blumm et al.^13^, the recent paper by Iñiguez et al^5^. also builds a microscopic model of list dynamics but differs from the former in two aspects: First, Iñiguez et al^5^. study open lists where (like many real-world rankings) only the top elements are explicitly ranked. Second, they do not assume the ranking is based on a variable associated with the items. The model of Iñiguez et al^5^. consists only of replacement (where an out-of-list item substitutes an element) and displacement (where an element moves within the list and offsets the others). The universality in their model refers to a non-trivial consistency relation that the rates of replacement and displacement must obey, which they furthermore corroborate with empirical data. Details of the displacement dynamics do not matter, and the relation holds whether the jumps in the list follow a fat-tailed distribution or not.

## Future outlook

Discovering universal relations of simple observables in list dynamics has more practical benefits than the lofty goals of Kant and others. They give us a more precise language to describe a phenomenon. Thanks to Iñiguez et al., we know the replacement rate is sufficient to explain the state of ranking dynamics. Thanks to Blumm et al., we can describe an item in a ranking just by the phase to which it belongs.

Since rankings are everywhere, so should practical (commercial or otherwise) applications of the theory of ranking dynamics be. While it is hard to speculate about the exact form of such applications, highly predictable, persistent patterns—the statistical laws discussed above—would be valuable as foundations. Ref. ^5^ also opens several new research directions. First, the role of memory in real-world list dynamics seems to be uncharted territory. One would need more information than about the present to determine the evolution of many types of real-world lists—few people would predict an aged tennis player in decline to bounce back into the top tier. This is in stark contrast to the memory-less models of Refs. ^5^ and^13^. Second, because of their even more fundamental role for human organization, it would be interesting to study the dynamics of hierarchies (the big caveat, of course, being that there is no universally accepted definition of hierarchy^6^).

To fulfill the promises of complex systems science, we need to keep discovering fundamental relations governing simple phenomena such as ranking dynamics. Even though not being theories of everything (as was the ambition of some complexity science in the 1990s^12^), such statistical laws are still the building blocks we need for the physics-type explanations in the social (and life) sciences that Kant and his contemporaries envisioned. Whether or not discoveries like those of Iñiguez et al. can also lead to forms of social organization where such natural laws complement juridical ones—as Enlightenment thinkers also believed—remains to be seen.
